# Polymorphic microsatellite markers demonstrate hybridization and interspecific gene flow between lumbricid earthworm species, *Eisenia andrei and E*. *fetida*

**DOI:** 10.1371/journal.pone.0262493

**Published:** 2022-02-18

**Authors:** Marta Jaskulak, Agnieszka Rorat, Franck Vandenbulcke, Maxime Pauwels, Paweł Grzmil, Barbara Plytycz

**Affiliations:** 1 Laboratoire de Génie Civil et géo-Environnement, Univ. Lille, IMT Lille Douai, Univ. Artois, Yncrea Hauts-de-France, ULR4515—LGCgE, Lille, France; 2 Univ. Lille, CNRS, UMR 8198 - EEP - Laboratoire Evolution Ecologie Paléontologie F-59000 Lille, France; 3 Department of Immunobiology and Environment Microbiology, Faculty of Health Sciences with Institute of Maritime and Tropical Medicine, Medical University of Gdansk, Gdansk, Poland; 4 Laboratory of Genetics and Evolutionism, Institute of Zoology and Biomedical Research, Jagiellonian University, Krakow, Poland; 5 Department of Evolutionary Immunology, Institute of Zoology and Biomedical Research, Jagiellonian University, Krakow, Poland; Jeju National University, REPUBLIC OF KOREA

## Abstract

The lumbricid earthworms *Eisenia andrei* (Ea) and *E*. *fetida* (Ef) have been used as model organisms for studies on hybridization. Previously they have been identified by species specific sequences of the mitochondrial COI gene of maternal origin (‘a’ or ‘f’) and the nuclear 28S gene of maternal/paternal origin (‘A’ or ‘F’). In experimental crosses, these hermaphroditic species produce progeny of genotypes Ea (aAA), Ef (fFF) and hybrids (aAF and fFA) originating by self-fertilization or cross-fertilization. To facilitate studies on new aspects of the breeding biology and hybridization of earthworms, polymorphic microsatellite markers were developed based on 12 Ea and 12 Ef specimens and validated on DNA samples extracted from 24 genotyped specimens (aAA, fFF, aAF and fFA) from three laboratory-raised families and 10 of them were applied in the present study. The results indicate that microsatellite markers are valuable tools for tracking interspecific gene flow between these species.

## Introduction

The uniformly reddish *Eisenia andrei* (Ea) and striped ‘tiger worms’ *E*. *fetida* (Ef) are widely used in toxicology, ecotoxicology, ecology, environmental studies [1, 2] and in biomedicine as a source of bioactive molecules [3, 4], all of which require accurate species identification. However the existence of hybrids was revealed by mixed patterns of electromorphs of esterase activity in laboratory mated specimens [5] and then relicts of past hybridization between Ea and Ef were recognized in natural populations from Scandinavia [6]. Asymmetrical hybridization has been experimentally proven in the progeny of laboratory-paired Ea and Ef virgin specimens delimited by species-specific sequences of the mitochondrial COI gene of maternal origin and the 28s rRNA gene of maternal/paternal origin [7, 8]. The first generation offspring of these hermaphroditic species included self-fertilized Ea and Ef specimens and fertile hybrids derived from Ea ova fertilized by Ef spermatozoa, while sterile hybrids from Ef ova fertilized by Ea spermatozoa appeared only among progeny of back-crossed Ea-derived hybrids with Ef parental specimens [7–10]. Although the reproductive capabilities of hybrids was gradually impaired [9], interspecies gene flow was documented by intermediate pigmentation patterns and by the presence of Ea-specific fluorophore not only in hybrids but also in a few Ef specimens [4, 8]. Novel species-specific genetic markers are needed for detailed studies on mechanisms of hybridization and gene introgression in these convenient model species.

Microsatellites are highly polymorphic, very short nucleotide sequences inherited in a codominant way and repeated many times in the nuclear genome [11] that can be used for addressing questions concerning the mating systems, genetic diversity, and population structure of a diverse array of organisms [12]. Since next-generation sequencing (NGS) has become more and more accessible, the identification and characterization of species-specific microsatellites is currently faster and requires less effort.

Polymorphic microsatellite markers have been developed for several earthworm species, including *Aporrectodea longa* [13], *Aporrectodea icterica* [14, 15], *Allolobophora chlorotica* [16, 17], *Hormogaster elisae* [18], *Dendrobaena octaedra* [11], *Amynthas cortices* [19], several *Lumbricus sp*. [12, 20–25], *Drawida gisti* [26], and *Eisenia fetida* [27], but not for *E*. *andrei*.

Thus, in response to the continuously growing need for new species-specific markers for studies on hybridization, the aim of the present work was to develop polymorphic microsatellite markers for *E*. *fetida* and *E*. *andrei* followed by their validation on DNA samples from three families of earthworms genotyped during previous experiments. Our results indicate that microsatellite markers are valuable tools for tracking interspecific gene flow.

## Materials and methods

### Earthworms

Lumbricid earthworms *Eisenia andrei* (Ea) and *E*. *fetida* (Ef) originally derived from laboratory stocks at the University in Lille (France) were cultured for a decade in the laboratories of the Institute of Zoology and Biomedical Research of the Jagiellonian University (Krakow, Poland), and in parallel for the last five years in the College of Natural Sciences, University of Rzeszów [7, 9, 10, 28, 29]. For several years they were used as convenient models for studies of hybridization of these simultaneous hermaphrodites by genotyping them by species-specific sequences of mitochondrial COI gene (‘a’ for Ea and ‘f’ for Ef) and diploid nuclear sequences of 28s rRNA gene of maternal/paternal origin (‘A’ for Ea and ‘F’ for Ef) as aAA, fFF and aAF or fFA interspecific hybrids [7–10]. Every genotyped specimen from these experiments was marked by the genetic symbols followed by a unique numerical code and all sequences have been deposited in GeneBank [7, 9, 10, 28] while ethanol-fixed posterior segments were preserved for further use.

Twelve adult specimens of each species, Ea and Ef, derived from Lille, Krakow and Rzeszow laboratory cultures were chosen to create two separate pools of DNA for microsatellite library preparation. Then another 15 specimens per species were used to establish numbers of alleles of potential microsatellite markers. Finally DNA samples from ethanol-fixed posterior segments of 24 earthworms from previous laboratory studies on hybridization [7, 9] were used for a pilot application of microsatellite markers in tracking interspecific gene flow between Ea and Ef.

### DNA extraction, enrichment and microsatellite library construction

Genomic DNA was extracted from supravitally amputated tail tips (approx. 50 mg of tissue). Homogenisation was performed by freezing the tail in liquid nitrogen, followed by mechanical homogenisation with a mortar. Afterwards, the genomic DNA was extracted with Universal DNA/RNA/Protein kit (Eur_x_, Poland), following the manufacturer’s instructions. The quality and quantity of the extracted DNA were checked on the SPECTROstar Nano spectrometer (BMG LABTECH, Germany). Prior to the NGS sequencing, the quality and quantity of extracted DNA were rechecked using a 2100 Bioanalyzer (Agilent Technologies, USA). 100 ng of DNA from each of 12 specimens per species was used to prepare two pools of DNA (one for Ea and one for Ef). The samples were then sent to the GenoScreen company (Lille, France), which prepared a microsatellite-enriched genomic library as described previously [30]. Both pools of DNA were sequenced with Illumina MiSeq flowcell v2 2x250 bp paired-end sequencing kit. Total DNA was mechanically fragmented, and enrichment was performed with probes containing 8 microsatellite motifs: TC TG, ACG, AGG, AAC, AAG, ACTC, ACAT. The QDD v.3 software [31] was used for microsatellite identification from the raw sequences, including all bioinformatics steps from adapter removal from raw sequences, detection of microsatellites, detection of redundancy/possible mobile element association, selection of sequences with target microsatellites and PCR primer design using BLAST (https://blast.ncbi.nlm.nih.gov/Blast.cgi), ClustalW [32] and Primer3 [33] programs. Only perfect di/tri/tetra motifs were kept, with A and B designs (according to QDD internal parameters: regions that do not have multiple microsatellites, nanosatellite, and homopolymers), and a minimum of 20 bp between the primer and the microsatellite.

### PCR and data analysis

The PCR reactions were performed in a 10 μL reaction volume (~100 ng genomic DNA, 1X PCR buffer, 6 pmol dNTPs, 37.5 pmol MgCl_2_, 0.5 U Taq DNA polymerase, 5 pmol of the forward primer, and 5 pmol of the reverse primer), performed in a Veriti thermal cycler (Applied Biosystems). The thermal profile of the amplification for all markers had an initial denaturation at 95°C for 10 min, followed by 40 cycles of: 30 s at 95°C, 30 s at 55°C and 60 s at 72°C, with a final extension of 10 min at 72°C. Amplicons were separated using automated high-resolution capillary electrophoresis (QIAxcel^®^ —Pure Excellence, Qiagen, Germany).

Alleles were sized using a 50–350 bp standard (LI-COR Biosciences), and genotypes were scored using SAGA v.3.3 software (LI-COR Biosciences). The species-specific primer sets were also checked for cross-species amplification under the same conditions as described above.

## Results and discussion

### Development of microsatellite markers for Ea and Ef

Genetic loci containing simple sequence repeats (SSRs, i.e. microsatellites) can be used as powerful markers in population genetics primarily because they are found throughout the nuclear genome, generally have several alleles per locus, and are inherited in a codominant way [11].

A total of 1 709 140 reads obtained for Ef were assembled into 918733 contigs, whereas 2 094 624 reads obtained for Ea were assembled into 1 140 654 contigs. Overall, 19 758 pairs of primers were designed for Ef and 23 255 for Ea. After that, 3 777 primer pairs for Ef and 4 258 primer pairs for Ea were validated with QDD v3 software. Primer sequence, product size and the sequence of each product are available elsewhere [34].

Out of those, 47 primer pairs per each species, characterized by the largest repeat number, were selected for amplification and polymorphism tests. In samples of Ef, 36 out of 47 loci amplified reliably and showed evidence of polymorphism. In samples of Ea, 30 out of 47 loci amplified reliably and showed evidence of polymorphism. These markers were initially tested using 15 Ea and 15 Ef individuals to assess the number of alleles for each marker. Ten microsatellite markers demonstrating the largest variability (5 for Ea and 5 for Ef) were used in the present study because of their potential usefulness in the discrimination between Ea, Ef and hybrid specimens. Their primer sequences, repeat motif, number of alleles, and GenBank accession numbers for sequences are shown in Table 1.

**Table 1:** Characterization of microsatellite loci isolated from *E*. *andrei* (a) *and E*. *fetida* (f) used in present paper.

marker	Primer sequence	Repeat motif	Size range (bp)	Dye	N_A_	Allele size range	Most frequent allele size	GenBank Accession number
**a1**	ACCATTGAGTGTAAATACATTACTCCA AGCGCTTATGCAGTGTATTTAGT	(AAT)17	173	6FAM	6	143–180	180	MW521367
**a3**	AGCAGAAGATTTGAGGCGGT CTGAGACATCTGCCAGCGAA	(AATG)15	177	VIC	3	143–184	161	MW521370
**a5**	CACCACGAGTACCAGCTGAA ACGTCATTGGGTTTAATTATCACCT	(ATC)12	171	PET	5	169–193	169	MW521372
**a6**	CGCTTTGTTCCAGAAGCTGC GCACAGGATATGCTTCAATTGCT	(AATG)11	200	PET	4	193–209	197	MW521373
**a7**	TTTAGAACAGAAATTGAAAGACCGA GCTGGCTTACAATTGCACCA	(AC)11	147	VIC	4	148–166	166	MW521374
**f3**	CTAATAGTGTGATGTGGCTGCC CAACATCCACAACCCATCTGT	(AATC)16	240	NED	8	203–284	203	MW521357
**f4**	TCCCTCTGCGTTTCTGACAG CCCAAGCTGGCATGCAAT	(ATC)16	187	6FAM	4	164–194	194	MW521358
**f6**	ACTGTATCCGTGCATGGCAT CCAATAATCAATTTCTAGAGGTTCCA	(AC)15	185	NED	4	178–202	178	MW521360
**f7**	ACATCTACGTCACTAGCCCTCT ACTGTTGAACTGAATGCGCTG	(ATC)12	291	NED	3	265–297	297	MW521362
**f9**	TGATGACAATAATATTGATGGTGACTT AACACTCGAAGAAGGTTAAGCAC	(AGG)11	140	6FAM	5	124–162	124	MW521365

[Repeat motif–repeat motif with a number of repeats, N_A_—number of alleles in analyzed population].

### Efficacy of microsatellites for investigation of hybridization and gene flow

#### Offspring of Ea+Ef pair

**Fig 1:**
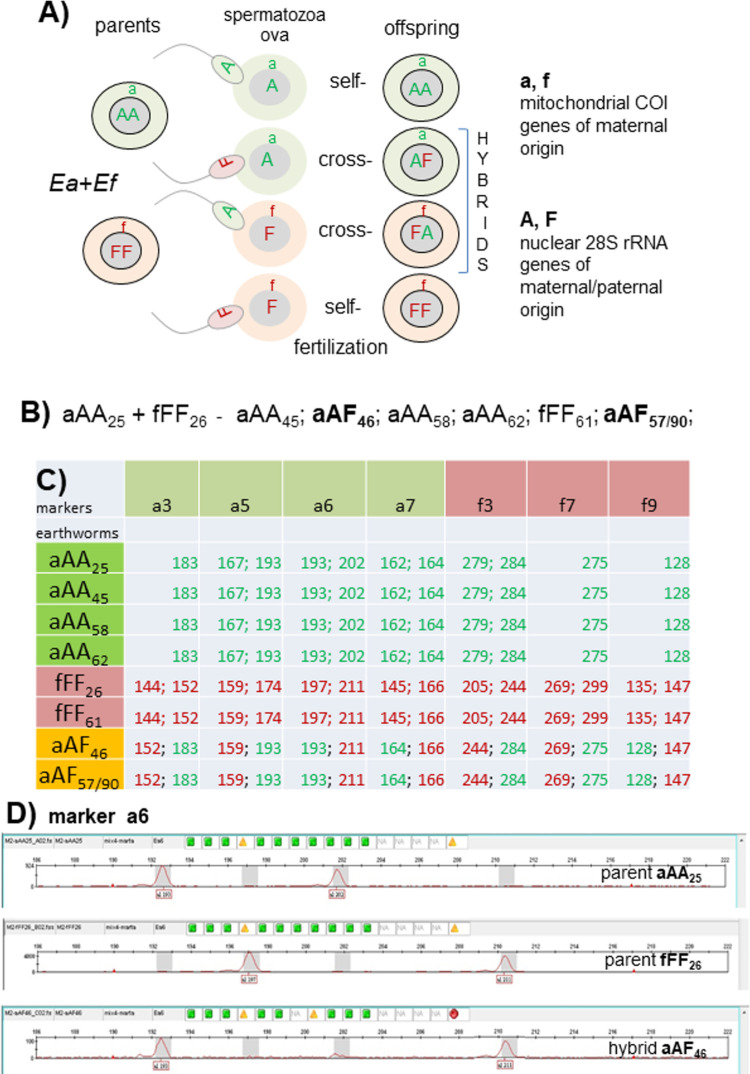
Scheme of mating of EaxEf earthworm pairs. (A) The germline cells of parental earthworms aAA and fFF, their gametes (‘aA’ or ‘fF’ ova and ‘A’ or ‘F’ spermatozoa) and self-fertilization or cross-fertilization resulting in zygotes of the pure Ea (aAA) and Ef (fFF) species or their Ea-derived aAF or Ef-derived fFA hybrids (the latter were lacking in our experiments); (B) Offspring of aAA+fFF earthworms from previous experiments; each earthworm marked by genotype followed by unique numerical code [7]; (C) Data concerning selected microsatellite markers (numbers indicate allele sizes) in particular members of this family; (D) Examples of visualization of a6 microsatellite marker sizes of the parental specimens aAA25 and fFF26 and hybrid aAF46. The Y-axis shows fluorescence intensity of PCR products while the X-axis shows the size of particular alleles.

*Eisenia* sp. earthworms are simultaneous hermaphrodites, thus each specimen produces ova and spermatozoa. In contrast to Ea and Ef from Spain, capable of uniparental reproduction by self-fertilization [35], these species from French, Polish, and Hungarian laboratory stocks are unable to self-fertilize when cultured in isolation but the presence of conspecific or closely related partner (like Ea, Ef and their hybrids) induces copulatory behavior [10]. As described earlier [7], copulating partners exchange sperm that moves from the male pores along the external body grooves to the partner’s spermatheca; at this stage, spermatozoa from a single individual may be admixed with the partner’s spermatozoa and thus both auto- and allo-spermatozoa may be stored in spermathecas of copulating partners. Earthworms separate after copulation and after several days the clitellum of each individual produces a mucous tube forming an oval capsule (cocoon) harvesting the individual’s ova and both auto- and allo-spermatozoa, thus ova within the same cocoon may be either self-fertilized or cross-fertilized. Thus, the progeny of interspecific Ea+Ef pairs is expected to have both a new generation of pure Ea or Ef specimens and interspecific hybrids derived either from Ea ova fertilized by Ef spermatozoa (aAF), and Ef ova fertilized by Ea spermatozoa (fFA) (Fig 1A). Contrary to such expectations, over a 5-year experiment using laboratory pairings of virgin Ea and Ef partners, first generation offspring included relatively common aAA, fFF, and aAF specimens, whereas fFA hybrids were not detected [7–10], as exemplified in Fig 1B.

In this paper, ethanol-fixed samples from the parental specimens aAA25+fFF26 and some of their offspring from experiments performed in 2018 [7] were analyzed using seven microsatellite markers, i.e. a3, a5, a6, a7,f3, f7, f9 (Fig 1C) visualized on an example of the marker a6 on Fig 1D). The results were fully concordant with data obtained using the COI/28S markers, showing that three aAA offspring share all microsatellites with the aAA25 parent, one fFF offspring shares alleles with the fFF26 parent, and two offspring specimens (aAF46 and aAF57/90) are interspecific hybrids possessing one nuclear allele from Ea and the second from Ef. It is worth noting that microsatellite analysis cannot clearly determine whether the hybrids were derived from Ea or Ef ova, thus COI gene was necessary to discriminate this.

#### Offspring of back-cross aAF+aAA

**Fig 2:**
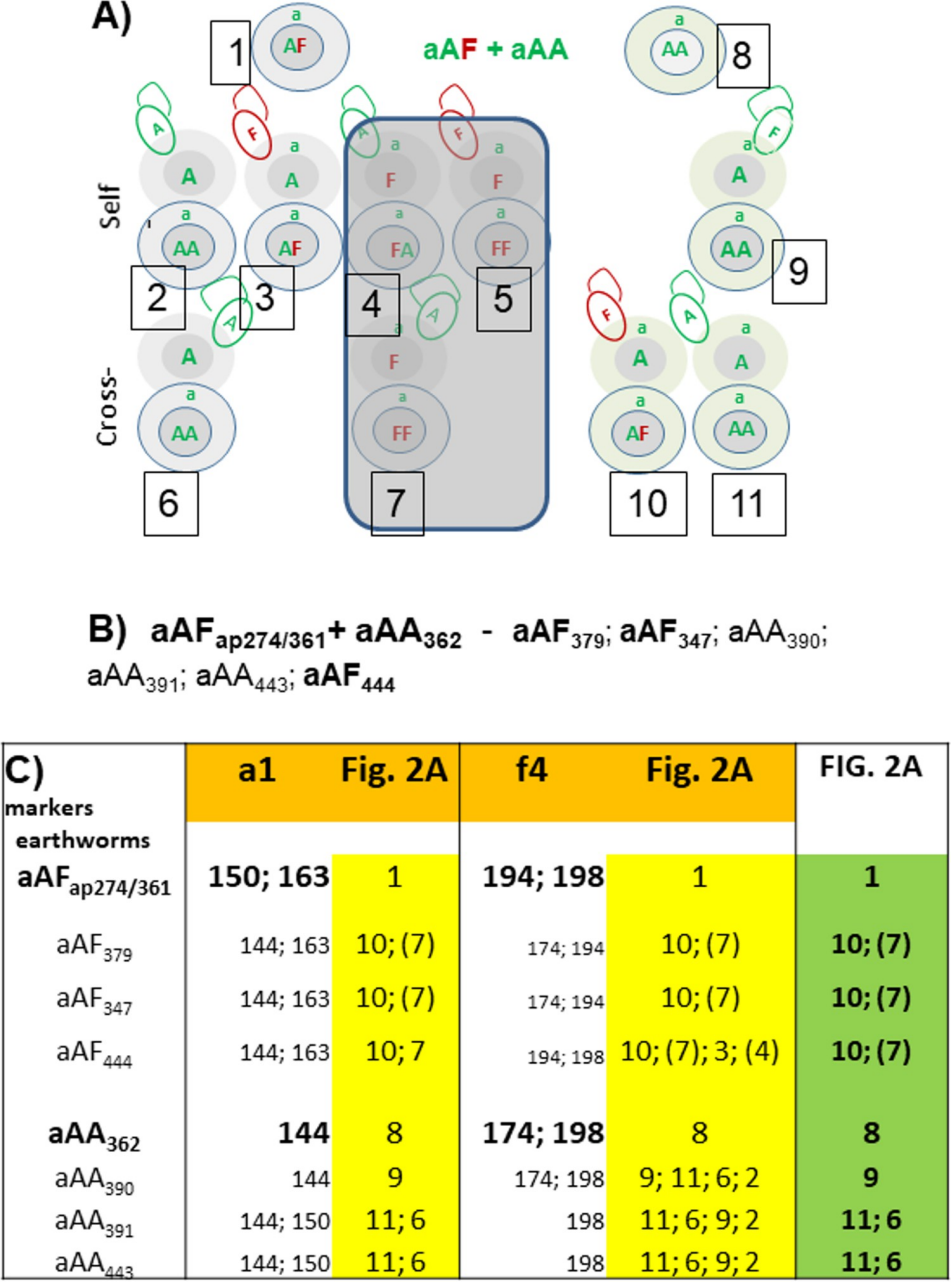
Considerations on the results of mating the aAF hybrid with aAA parental specimen. (A) Back-cross of aAF+aAA pair; symbols as on Fig 1. (B) Offspring of pair aAF+aAA (the example taken from [9]. (C) Allele sizes of a1 and f4 microsatellite markers in particular specimens from the above family and putative identification with genotypes 1–11 in Fig 2A (in yellow for particular markers and in green as most probable).

Each aAF hybrid (numbered as 1 in Fig 2A) produces two kinds of ova, ‘aA’ and ‘aF’ and two kinds of spermatozoa, ‘A’, and ‘F’. Thus these hybrids may give four types of progeny: aAA, aAF, aFA, and aFF by self-fertilization (numbered 2–5 in Fig 2A). The aAA partner (number 8) produces only ‘aA’ ova and ‘A’ spermatozoa, thus may give the aAA offspring by self-fertilization (number 9). However, neither aAF nor aAA specimens reproduced in isolation but initiated production of fertile cocoons only in the presence of a closely related partner [10]. Cross-fertilized ‘aA’ ova of the Ea partner give pure Ea specimens (number 11) and aAF hybrid (number 10) (Fig 2A). Cross-fertilized ‘aA’ ova of the hybrid give aAA specimens (number 6), while ‘aF’ ova give aFA hybrids (number 7). However, ‘aF’ ova may be incompatible due to cyto-nuclear mismatch, and their vitality might be strongly impaired [36–38], thus mitonuclearly incompatible ova and potentially incompatible resulting fertilized cells are shadowed (numbers 4, 5, and 7 on Fig 2A).

The offspring of aAFap274/361+aAA362 pair from previous experiments [9] encompassed both ‘pure’ Ea specimens and the next generation of Ea-derived hybrids (Fig 2B). Each of the pure specimens may be derived by four different genotype combinations (numbers 2; 6; 9; 11), while each hybrid by another four different combinations (numbers 3; 4; 7; 10) (Fig 2A). The microsatellites applied here have helped reduce the numbers of such possibilities (Fig 2C). Microsatellite marker a1 is heterozygous in parental aAF274/361 hybrid (with alleles 150 and 163) while a1 is homozygous in aAA362 parent (allele 144 only). All investigated hybrid offspring (aAF379, aAF347 and aAF444) are heterozygotes with allele 144 inherited from Ea and 163 from the hybrid. This combination of alleles fits the examples numbered as 10 and 7 (the latter less probable) in Fig 2A.

Among Ea offspring of the investigated pair, only aAA390 specimen originated by self-fertilization of the Ea parent as they share allele 144 of marker a1 (9 from Fig 2A). The two remaining aAA391 and aAA443 specimens inherited allele 144 from the Ea parent and allele 150 from the hybrid, as illustrated by 11 in Fig 2A. However it cannot be excluded that allele 150 was inherited from the hybrid and allele 144 from the Ea parent, as illustrated by 6 in Fig 2A.

The same family was analyzed by the marker f4 (Fig 2C) that is heterozygous in both parents, with allele 198 being shared by both of them, and the results were even less discriminatory as the origin of all Ea offspring and aAF444 hybrid might be explained by the four possibilities. However, the possible combinations revealed by f4 marker were also shared with the more discriminatory marker a1, thus the latter were considered as most probable for this family (last green column of Fig 2C). Thus the marker a1 was much more valuable for investigation of this particular family than the f4 marker, but further markers are needed to precisely discriminate the origins of some particular earthworms, including discerning between possibilities 6 or 11.

The presence of microsatellite marker (e.g. allele 150 of a1 marker) both in the hybrid parent (1) and in the aAA genotype numbered as 11 originating from Ea-derived ova fertilized by hybrid-derived spermatozoa is indicative of gene transfer from Ef to Ea. This might be exemplified by hypothetically polymorphic genes responsible for Ef-specific striped body pigmentation pattern of the ‘tiger’ fFF grandparent mated with aAA earthworm, through the aAF parents (1) to some Ef-like earthworms (11) with mixed pigmentation pattern but genotyped as aAA by species-specific sequences of COI/28S genes [7, 8]

#### Offspring of back-cross aAF+fFF

**Fig 3:**
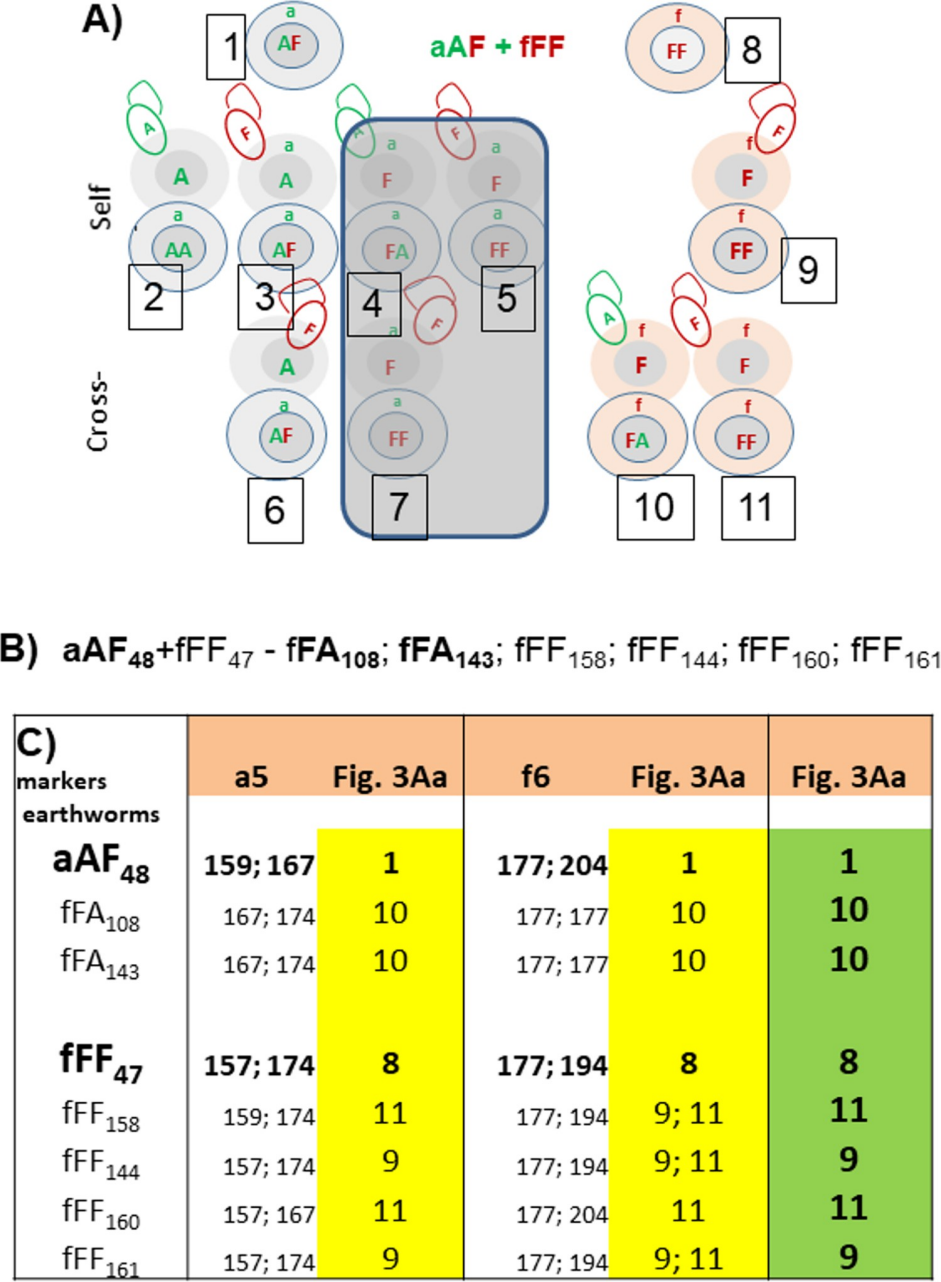
Considerations on the results of mating the aAF hybrid with fFF parental specimen. (A) Back-cross of aAF+fFF pair; symbols as on Fig 1. (B) Offspring of aAF+fFF pair (the example from [7]. (C) Allele sizes of a5 and f6 microsatellite markers in particular specimens from the above family and putative identification with genotypes numbered 1–11 on Fig 3A (in yellow for particular markers and in green as most probable).

Among offspring of back-cross aAF+fFF (Fig 3A), cross-fertilized ova of aAF hybrids may give a new generation of aAF hybrids (6) and (unique) mitonuclearly-incompatible aFF specimens (number 7) very rare in our previous experiments [9] while cross-fertilized ‘fF’ ova of the fFF specimens can give fFA hybrids fertilized by ‘A’ spermatozoa from the aAF parent (number 10) and fFF specimens (number 11) fertilized by hybrid’s ‘F’ spermatozoa (Fig 3A). Such family is exemplified here by progeny of aAF48+fFF47 specimens from previous experiments [7] that gave two Ef-derived hybrids fFA and four fFF specimens (Fig 3B) among offspring. Analysis of distribution of a5 and f6 alleles was fully consistent with the only possibility of the origin of Ef-derived fFA hybrids, i.e. cross-fertilization of ‘fF ova by aAF-derived ‘A’ spermatozoa (10) while new generation of aAF hybrids (6) was absent among progeny of this family (but they were present among offspring of other back-crossed aAF+fFF pairs from previous studies [9]).

There are two possibilities of the origin of fFF specimens among offspring of an aAF+fFF pair, i.e. either self-fertilization (9) or cross-fertilization of ‘fF’ ova by hybrid-derived ‘F’ spermatozoa (11). The a5 microsatellite marker, being heterozygous in both parental species, has shown unequivocally that fFF144 and fFF161 originated by self-fertilization (9) while fFF158 and fFF160 came from cross-fertilization of ‘fF’ from the fFF parent by hybrid-derived ‘F’ spermatozoa (11). In the case of the heterozygous f6 marker, allele 177 was present in all members of this family thus more genotypic combinations were possible, but only those shared by both markers (a5 and f6) are considered a likely explanation of the origin of fFF offspring (last column of Fig 3C).

These results might shed more light on the transfer of putative genes responsible for fluorescence of coelomic fluid, being a fingerprint of aAA specimens [39]. Fluorescence is present in most aAF hybrids and in further backcrosses with fFF specimens (devoid of fluorescence), it seems to be acquired by rare fFA and fFF earthworms [4, 8], a phenomenon worthy of additional investigation.

### Microsatellite markers and earthworm reproduction

Application of microsatellite markers to laboratory-paired virgin aAA+fFF, aAF+aAA and aAF+fFF earthworms and their progeny have fully confirmed previously postulated genotypic combinations of their offspring [7–10]. Moreover, we demonstrate that self-fertilization is not the only pathway for the production of a new generation of pure aAA and fFF specimens, since cross-fertilization of parental specimens significantly contributes to this process. We show that cross-fertilization resulted in interspecific gene flow from pure-species grandparents (P generation) through interspecific fertile aAF hybrids (F1 generation) to ‘pure’ aAA or FF specimens (F2 generation), inheriting the grandparents’ markers. This genotyping strategy might provide a means for linking some of microsatellite markers with physiologically important genes and, i. e. help to explain previously described inheritance patterns of body pigmentation or production of some fluorescent markers [4, 7, 8]. On the other hand, Novo et al. [18] revealed that microsatellite markers are a valuable tool for studies on paternity of the earthworm *Hormogaster elisae* after copulation with three successive partners. Microsatellite markers applied to field-sampled earthworms from various localities might reveal signs of past hybridization and thus shed more light on the evolution of these species [6, 16].

In conclusion, the novel polymorphic microsatellite markers for *E*. *fetida* and *E*. *andrei* are potentially useful for the analysis of genetic diversity, population structure and dispersal including past and present hybridization events in natural populations and are valuable in laboratory studies on the controlled reproduction and gene flow between these two hermaphroditic species. *E*. *fetida* and *E*. *andrei* may be considered as potential ring species [40] attractive for studies on speciation within the *Eisenia* complex [41, 42] due to their capacity for hybridization, which is increasingly being appreciated as a potent driving force of evolution [e.g. 43].

## References

[1] Ding W, Li Z, Qi R, Jones DL, Liu Q, Liu Q, et al. Effect thresholds for the earthworm *Eisenia fetida*: Toxicity comparison between conventional and biodegradable microplastics. Sci Total Environ. 2021; 781:146884. 10.1016/j.scitotenv.2021.146884

[2] Rybak AV, Belykh ES, Maystrenko TA, Shadrin DM, Pylina YI, Chadin IF, et al. Genetic analysis in earthworm population from area contaminated with radionuclides and heavy metals. Sci Total Environ. 2020; 723:137920. doi: 10.1016/j.scitotenv.2020.137920 . 32213403

[3] Roubalová R, Płytycz B, Procházková P, Navarro Pacheco NI, Bilej M. Annelida: Environmental Interactions and Ecotoxicity in Relation to the Earthworm Immune System. In Cooper E. (eds) Advances in Comparative Immunology. Springer Cham. 2018; 933–951. 10.1007/978-3-319-76768-0_27

[4] Kruk J, Dziurka M, Płytycz B. Identification of new fluorophores in coelomic fluid of *Eisenia andrei* earthworms. PLOS ONE. 2019; 14: e0214757. doi: 10.1371/journal.pone.0214757 . 30921437 PMC6438515

[5] Oien N, Stenersen J. Esterases of earthworms–III. Electrophoresis reveals that Eisenia fetida (Savigny) is two species. Comparative Biochemistry and Physiology Part C: Comparative Pharmacology. 1984; 78:277–282. doi: 10.1016/0742-8413(84)90083-5 . 6207979

[6] Martinsson S, Erséus C. Hybridisation and species delimitation of Scandinavian *Eisenia* spp. (Clitellata: Lumbricidae). Eur J Soil Biol. 2018; 88:41–47. 10.1016/j.ejsobi.2018.06.003

[7] Plytycz B, Bigaj J, Osikowski A, Hofman S, Falniowski A, Panz T, et al. The existence of fertile hybrids of closely related model earthworm species, *Eisenia andrei* and *E*. *fetida*. PLOS ONE. 2018; 13: e0191711. doi: 10.1371/journal.pone.0191711 . 29370238 PMC5784991

[8] Plytycz B, Bigaj J, Panz T, Grzmil P. Asymmetrical hybridization and gene flow between *Eisenia andrei* and *E*. *fetida* lumbricid earthworms. PLOS ONE. 2018; 13:e0204469. doi: 10.1371/journal.pone.0204469 . 30240427 PMC6150523

[9] Plytycz B, Bigaj J, Rysiewska A, Osikowski A, Hofman S, Podolak A, et al. Impairment of reproductive capabilities in three subsequent generations of asymmetric hybrids between *Eisenia andrei* and *E*. *fetida* from French, Hungarian and Polish laboratory colonies. PLoS One. 2020; 15 e0235789. doi: 10.1371/journal.pone.0235789 . 32645117 PMC7347191

[10] Podolak A, Kostecka J, Hofman S, Osikowski A, Bigaj J, Plytycz B. Annual Reproductive Performance of *Eisenia andrei* and *E*. *fetida* 2 in Intra- and Inter-Specific Pairs and Lack of Reproduction of Isolated Virgin Earthworms. Folia Biol. 2020; 68:1–6. 10.3409/fb_68-1.01

[11] Mustonen M, Haimi J, Kesäniemi J, Högmander H, Knott KE. Variation in gene expression within clones of the earthworm *Dendrobaena octaedra*. PLOS ONE. 2017; 12:e0174960. doi: 10.1371/journal.pone.0174960 . 28384196 PMC5383104

[12] Dupont L, Pauwels M, Dume C, Deschins V, Audusseau H, Gigon A, et al. Genetic variation of the epigeic earthworm *Lumbricus castaneus* populations in urban soils of the Paris region (France) revealed using eight newly developed microsatellite markers. Appl Soil Ecol. 2019; 135:33–37. 10.1016/j.apsoil.2018.11.004

[13] Strunk H, Hochkirch A, Veith M, Hankeln T, Emmerling C. Isolation and characterization of eleven polymorphic microsatellite markers for the earthworm *Aporrectodea longa* (Ude). Eur J Soil Biol. 2012; 48:56–58. http://dx.doi.org/10.1016%2Fj.ejsobi.2011.11.004

[14] Torres-Leguizamon M, Mathieu J, Livet A, Decaëns T, Dupont L. Isolation of polymorphic microsatellite markers in *Aporrectodea icterica* (Savigny 1826). Soil Biol Biochem. 2012; 51:16–19. 10.1016/j.soilbio.2012.03.020

[15] Torres-Leguizamon M, Mathieu J, Decaëns T, Dupont L. Genetic Structure of Earthworm Populations at a Regional Scale: Inferences from Mitochondrial and Microsatellite Molecular Markers in *Aporrectodea icterica* (Savigny 1826). PLoS ONE. 2014; 9:e101597. doi: 10.1371/journal.pone.0101597 . 25003795 PMC4086927

[16] Dupont L, Lazrek F, Porco D, King A, Rougerie R, Symondson W, et al. New insight into the genetic structure of the *Allolobophora chlorotica* aggregate in Europe using microsatellite and mitochondrial data. Pedobiologia, 2011; 54:217–224 10.1016/j.pedobi.2011.03.004

[17] Dupont L, Porco D, Symondson WOC, Roy V. Hybridization relicts complicate barcode based identification of species in earthworms. Molecular Ecology Resources. 2016; 16: 883–894. doi: 10.1111/1755-0998.12517 26929276

[18] Novo M, Fernández R, Granado-Yela C, Gutiérrez López M, Díaz Cosín DJ. Does the order of copulation matter? Experimental paternity analyses in the earthworm *Hormogaster elisae* (Annelida: Hormogastridae). Pedobiologia. 2013; 56:97–104. 10.1016/j.pedobi.2013.01.002

[19] Cunha L, Thornber A, Kille P, Morgan AJ, Novo M. A large set of microsatellites for the highly invasive earthworm *Amynthas corticis* predicted from low coverage genomes. Appl Soil Ecol. 2017;119: 152–155. 10.1016/j.apsoil.2017.05.029

[20] Harper GL, Cesarini S, Casey SP, Morgan AJ, Kille P, Bruford MW. Microsatellite markers for the earthworm *Lumbricus rubellus*. Mol Ecol Notes. 2006; 6:325–327. 10.1111/j.1471-8286.2005.01219.x

[21] Lounsberry ZT, Ungerer MC, Snyder BA. Identification of 12 EST-derived SSR markers in *Lumbricus rubellus*. Pedobiologia. 2013;56(4–6): 191–193. doi: 10.1007/s11033-020-05799-4 . 32929651

[22] Anderson C, Cunha L, Sechi P, Kille P, Spurgeon D. Genetic variation in populations of the earthworm, *Lumbricus rubellus*, across contaminated mine sites. BMC Genet. 2017; 18:97. doi: 10.1186/s12863-017-0557-8 . 29149838 PMC5693503

[23] Klein A, Cameron EK, Heimburger B, Eisenhauer N, Scheu S, Schaefer I. Changes in the genetic structure of an invasive earthworm species (*Lumbricus terrestris*, Lumbricidae) along an urban–rural gradient in North America. Appl Soil Ecol. 2017; 120:265–272. doi: 10.1016/j.apsoil.2017.08.009 . 29176926 PMC5699645

[24] Souleman D, Grumiaux F, Frérot H, Vandenbulcke F, Pauwels M. Isolation and characterization of eight polymorphic microsatellites markers for the earthworm *Lumbricus terrestris*. European Journal of Soil Biology. 2016; 74:76–80. http://dx.doi.org/10.1016%2Fj.ejsobi.2016.03.009

[25] Audusseau H, Vandenbulcke F, Dume C, Deschins V, Pauwels M, Gigon A, et al. Impacts of metallic trace elements on an earthworm community in an urban wasteland: Emphasis on the bioaccumulation and genetic characteristics in *Lumbricus castaneus*. Sci Total Environ. 2020; 718:137259. doi: 10.1016/j.scitotenv.2020.137259 32105923

[26] Liu H, Zhang Y, Wang G, Chen J, Zhang Q, Ruan H. Development and characterization of microsatellite markers in the earthworm *Drawida gisti* Michaelsen, 1931 and cross-amplification in two other congeners. Mol Biol Rep. 2020; 47:8265–8269. doi: 10.1007/s11033-020-05799-4 32929651

[27] Somers CM, Neudorf K, Jones KL, Lance SL. Novel microsatellite loci for the compost earthworm *Eisenia fetida*: A genetic comparison of three North American vermiculture stocks. Pedobiologia. 2011; 54:111–117. 10.1016/j.pedobi.2010.11.002

[28] Rorat A, Kachamakova-Trojanowska N, Jozkowicz A, Kruk J, Cocquerelle C, Vandenbulcke F, et al. Coelomocyte-derived fluorescence and DNA markers of composting earthworm species. J Exp Zool A Ecol Genet Physiol. 2013; 321:28–40. doi: 10.1002/jez.1834 . 24115405

[29] Jaskulak M, Rorat A, Kurianska-Piatek L, Hofman S, Bigaj J, Vandenbulcke F, et al. Species-specific Cd-detoxification mechanisms in lumbricid earthworms *Eisenia andrei*, *Eisenia fetida* and their hybrids. Ecotoxicol Environ Saf. 2021; 208:111425. doi: 10.1016/j.ecoenv.2020.111425 . 33068978

[30] Malausa T, Gilles A, Meglécz E, Blanquart H, Duthoy S, Costedoat C, et al. High‐throughput microsatellite isolation through 454 GS‐FLX Titanium pyrosequencing of enriched DNA libraries. Mol Biol Rep. 2011; 11:638–644. doi: 10.1111/j.1755-0998.2011.02992.x . 21676194

[31] Meglécz E, Pech N, Gilles A, Dubut V, Hingamp P, Trilles A, et al. QDD version 3.1: a user-friendly computer program for microsatellite selection and primer design revisited: experimental validation of variables determining genotyping success rate. Mol Ecol Resour. 2014; 14:1302–1313. doi: 10.1111/1755-0998.12271 . 24785154

[32] Larkin MA, Blackshields G, Brown NP, Chenna R, McGettigan PA, McWilliam H, et al. Clustal W and Clustal X version 2.0. Bioinformatics. 2007; 23: 2947–2948. doi: 10.1093/bioinformatics/btm404 . 17846036

[33] Rozen S, Skaletsky H. Primer3 on the WWW for General Users and for Biologist Programmers. Bioinformatics Methods and Protocols. 2000; 132:365–386. doi: 10.1385/1-59259-192-2:365 . 10547847

[34] Jaskulak M, Vandenbulcke F, Rorat A, Pauwels M, Grzmil P, Plytycz B. Data on genome identification of microsatellite markers in *Eisenia fetida* and *Eisenia andrei–*DataInBrief, submitted. 10.1016/j.dib.2022.108612 PMC9679477 36425961

[35] Domínguez J, Velando A, Aira M, Monroy F. Uniparental reproduction of *Eisenia fetida* and *E*. *andrei* (Oligochaeta: Lumbricidae): evidence of self-insemination. Pedobiologia. 2003; 47: 530–534. 10.1078/0031-4056-00224

[36] Ma H, Marti Gutierrez N, Morey R, Van Dyken C, Kang E, Hayama T, et al. Incompatibility between Nuclear and Mitochondrial Genomes Contributes to an Interspecies Reproductive Barrier. Cell Metab. 2016; 24:283–294. doi: 10.1016/j.cmet.2016.06.012 . 27425585 PMC4981548

[37] Zhang C, Montooth KL, Calvi BR. Incompatibility between mitochondrial and nuclear genomes during oogenesis results in ovarian failure and embryonic lethality. Development. 2017; 144:2490–2503 doi: 10.1242/dev.151951 . 28576772 PMC5536873

[38] Hill GE. Mitonuclear Ecology. Oxford Scholarship Online. 2019. doi: 10.1093/oso/9780198818250.001.0001

[39] Albani JR, Demuynck S, Grumiaux F, Leprêtre A. Fluorescence fingerprints of Eisenia fetida and Eisenia andrei. Photochem Photobiol. 2003; 78: 599–602. 10.1562/0031-8655(2003)078<0599:ffoefa>2.0.co;2 14743869

[40] Blackmon H, Demuth JP. Ring species and speciation. In: eLS. John Wiley & Sons, Ltd: Chichester. 2012. doi: 10.1002/9780470015902.a0001751.pub3

[41] Payseur BA, Rieseberg LH. A genomic perspective on hybridization and speciation. Molecular Ecology. 2016; 25:2337–2360. doi: 10.1111/mec.13557 . 26836441 PMC4915564

[42] Runemark A, Vallejo-Martin M, Meier JI. Eukaryote hybrid genomes. PLoS Genetics. 2019; 15: e1008404. doi: 10.1371/journal.pgen.1008404 . 31774811 PMC6880984

[43] Pennisi E. Shaking up the tree of life. Science. 2016; 354:817–821. doi: 10.1126/science.354.6314.817 . 27856860

